# A bidirectional autoimmune cluster between vitiligo and rheumatoid arthritis: a large-scale population-based study

**DOI:** 10.1007/s00403-024-02965-7

**Published:** 2024-06-08

**Authors:** Naama Tova Cohen, Yochai Schonmann, Khalaf Kridin

**Affiliations:** 1 Azrieli Faculty of Medicine, Bar-Ilan University, Safed, Israel; 2 Medical School for International Health, Ben Gurion University, Negev Be’er Sheva, Israel; 3 Clalit Health Services, Tel-Aviv, Israel; 4 Unit of Dermatology and Skin Research Laboratory, Galilee Medical Center, Nahariya, Israel; 5 Lübeck Institute of Experimental Dermatology, University of Lübeck, Lübeck, Germany

**Keywords:** Vitiligo, Rheumatoid arthritis, Cohort study, Case-control study

## Abstract

A knowledge gap exists regarding the association between vitiligo and rheumatoid arthritis (RA) due to the absence of large-scale cohort studies designed to investigate this association. To investigate the bidirectional epidemiological association between vitiligo and RA. A population-based study was conducted using Clalit Health Services (CHS) database (2002–2019) using both a cohort study and a case-control study design. Adjusted hazard ratio (HR) and odds ratio (OR) were calculated by multivariate Cox and logistic regressions, respectively. Overall, 20,851 vitiligo patients and 102,475 controls were included. The incidence of new-onset RA was 4.1 (95% CI 3.0–5.4) and 2.9 (95% CI 2.4–3.3) cases per 10,000 person-years among patients with vitiligo and controls, respectively. Patients with vitiligo had a significantly increased risk of developing new-onset RA (adjusted HR, 1.44; 95% confidence interval [CI], 1.02–2.02, *P* = 0.036). The likelihood of having vitiligo was significantly elevated after a preexisting diagnosis of RA (adjusted OR, 1.67; 95% CI, 1.38–2.03; *P* < 0.001). Relative to the remaining patients with vitiligo, those with vitiligo and comorbid RA demonstrated an elevated risk of all-cause mortality (adjusted HR, 1.61; 95% CI, 1.03–2.51; *P* = 0.037). Our study confirms the bidirectional association between vitiligo and RA. Physicians treating patients with vitiligo should be aware of the association in clinical practice.

**Supplementary Information:**

The online version contains supplementary material available at 10.1007/s00403-024-02965-7.

## Introduction

Vitiligo is a chronic autoimmune skin disorder characterized by the destruction of melanocytes, resulting in depigmentation of skin regions [1]. The etiology of vitiligo is multifactorial involving genetic factors, environmental triggers, activation of inflammatory mediators, and an autoimmune response [2]. Recent advancements have led to the recognition of vitiligo as an immunological disorder, and vitiligo has been associated with various autoimmune-related disorders, including Hashimoto’s thyroiditis, Graves’ disease, type 1 insulin-dependent diabetes mellitus, and Addison’s disease, supporting the autoimmune theory [3]. This association may arise from shared autoimmune and genetic susceptibilities. Studies have shown that individuals with vitiligo have an increased incidence of other autoimmune conditions [4].

A recent genome wide association study (GWAS) has significantly advanced the understanding of the genetic basis of vitiligo. Various genetic loci and susceptibility genes have been identified, providing insights into the pathways involved in the pathogenesis of vitiligo [5].

Rheumatoid arthritis (RA) is a chronic autoimmune disease characterized by progressive joint damage and multi-system complications. RA is characterized by persistent inflammation which may result in long-lasting disability and increased mortality rate as compared to the general population [6]. There are various factors associated with the development of RA, including genetics, female sex, and environmental influences. The incidence of RA is higher in women, with females being two to three times more prone to the condition than males [7].

Both vitiligo and RA involve dysregulated immune responses, yet the pathogenesis involves distinct mechanisms. Dysregulation of cytokine activity is implicated in both conditions [8, 9]. Recently research examined the role of interleukin 27 (IL-27) that triggers Tr1 cells, which suppress the responses of Th2 and Th17 cells, which reduce the intensity of autoimmune diseases. Altered concentration in IL-27 have been observed in both RA and vitiligo emphasizing potential association [10–12]. Despite these common pathogenic themes, the epidemiological association of vitiligo with RA is yet to be firmly established.

In the current study, we sought to investigate the bidirectional associations between vitiligo and RA using a large-scale population-based study. We aimed to study the risk of new-onset RA among individuals with vitiligo and to calculate the odds of vitiligo occurring in patients who already have RA. Additionally, aimed to study determinants of RA among patients with RA.

## Methods

### Study design and data set

The current study aimed to investigate the bidirectional association between vitiligo and RA, a large-scale population-based study included two study designs. First, a retrospective cohort study design was used to longitudinally follow patients with vitiligo and estimate the incidence of new-onset RA. Second, a case-control study design was applied to estimate the prevalence of preceding RA (exposure) in patients with subsequent vitiligo (outcome). Considering the rare disease assumption, the latter design is likely to delineate the odds of vitiligo after RA.

We utilize the computerized data set of Clalit Health Services (CHS) as the data source. According to regulations of the National Health Insurance Law, residents in Israel are obligated to enroll in one of the four healthcare maintenance organizations. CHS is the largest healthcare provider organization in Israel, with 4,554,343 members as of 2018. CHS provides a comprehensive array of medical coverage, including both outpatient and inpatient settings. Each interaction within the healthcare system is recorded in the patient’s medical file, and the CHS database is continuously updated; the database offers an extensive overview of CHS enrollees over time, establishing its reliability as a source for epidemiological data.

### Study population and definition of covariates

The CHS database was screened for incident cases with a diagnostic code of vitiligo between the years 2002 and 2019. Patients were considered eligible for inclusion if one of the following criteria was fulfilled: (i) a documented diagnosis of vitiligo as registered by a board-certified dermatologist, or (ii) a diagnosis of vitiligo in discharge letters from dermatological wards. The diagnosis of RA was based on documentation by certified rheumatologists.

We enrolled a control group including up to five individuals without vitiligo per each case. Controls were matched based on age, sex, and ethnicity and were recruited on the day in which the corresponding case was diagnosed. Outcome measures were adjusted for demographic variables and putative confounding comorbidities, including smoking, diabetes mellitus, hypertension, hyperlipidemia, and body mass index (BMI).

### Statistical analysis

The comparison of variables between different comparator groups was performed using the chi-square test and t-test for categorical and continuous variables, respectively. In the cohort study design, incidence rates of RA were calculated for both vitiligo patients and controls and expressed as the number of events per 10,000 person-years. Hazard ratios (HR)s and 95% confidence intervals (CI)s for the risk of incident RA were found using the Cox regression model. Differences in the all-cause mortality of vitiligo patients with and without RA were estimated using a stratified log-rank test.

In the case-control study design, conditional logistic regression analysis was utilized to calculate odds ratios (ORs) and 95% CIs to compare cases and controls with reference to the presence of preexisting RA. Based on the temporal relationship between exposure and outcome in case-control studies, only individuals who developed vitiligo after the diagnosis of RA were included. Two-tailed P-values less than 0.05 were considered statistically significant. All statistical analyses were performed using SPSS software, version 25 (SPSS, Armonk, NY: IBM Corp).

## Results

### Characteristics of the study population

The study population included 20,851 patients with vitiligo and 102,475 age-, sex-, and ethnicity-matched controls. The mean (SD) age of patients with vitiligo was 34.7 (22.4) years; 10,570 (50.7%) patients were females, and 15,311 (73.4%) were of Jewish ancestry. The baseline characteristics of the study population are outlined in Table 1.

**Table 1:** Descriptive characteristics of the study population

Characteristic	Patients with vitiligo (*N* = 20,851)	Controls (*N* = 102,475)	*p*-value
*Age, years*			
Mean (SD)	34.7 (22.4)	34.6 (22.4)	0.608
Median (range)	32.4 (0.1–95.4)	32.4 (0.1–96.2)	
*Sex, n (%)*			
Male	10,281 (49.3%)	50,523 (49.3%)	0.991
Female	10,570 (50.7%)	51,952 (50.7%)	
*Ethnicity, n (%)*			
Jews	15,311 (73.4%)	75,249 (73.4%)	0.903
Arabs	5,540 (26.6%)	27,226 (26.6%)	
*BMI, mg/kg* ^*2*^			
Mean (SD)	25.2 (31.5)	24.8 (31.5)	0.241
Smoking, n (%)	5,207 (25.0%)	29,728 (29.0%)	< 0.001
Charlson comorbidity score, mean (SD)	0.4 (0.9)	0.4 (1.0)	0.876

*Abbreviations* N, Number; SD, standard deviation; BMI, body mass index

### The risk of developing RA among patients with vitiligo

In the retrospective cohort study design, patients with vitiligo were cumulatively followed for 115,210 person-years whereas controls were followed for 564,438 person-years. During the follow-up duration, 47 and 161 cases of new-onset RA occurred among patients with vitiligo and control individuals, respectively. Taken together, the incidence rates of RA were 4.1 (95% CI, 3.0–5.4) and 2.9 (95% CI, 2.4–3.3) cases per 10,000 person-years in patients with vitiligo and control individuals, respectively (Table 2).

**Table 2:** Incidence rates and hazard ratio of new-onset rheumatoid arthritis among patients with vitiligo (cohort study design)

	Vitiligo	Controls
Follow-up time, PY	115,210.2	564,438.1
Median follow-up time, years (range)	5.5 (0.1–14.5)	5.5 (0.1–14.5)
Number of events	47	161
Incidence rate per 10,000 PY (95% CI)	4.1 (3.0-5.4)	2.9 (2.4–3.3)
Unadjusted HR (95% CI) [p-value]	**1.43 (1.03–1.98) [0.031]**	Reference
*Sex- and age-stratified analysis*		
Male-specific HR (95% CI) [p-value]	**1.90 (1.07–3.39) [0.029]**	Reference
Female-specific HR (95% CI) [p-value]	1.27 (0.86–1.89) [0.233]	Reference
≥ 32.4 year-specific HR (95% CI) [p-value]	1.29 (0.90–1.86) [0.165]	Reference
< 32.4 year-specific HR (95% CI) [p-value]	**2.25 (1.06–4.74) [0.034]**	Reference
*Multivariate adjusted analysis*		
Age- and sex-adjusted HR (95% CI) [p-value]	**1.41 (1.02–1.95) [0.040]**	Reference
Fully adjusted HR (95% CI) [p-value] ^a^	**1.44 (1.02–2.02) [0.036]**	Reference

**Bold**: significant value

*Abbreviations* HR, hazard ratio; CI, confidence interval; PY, person-year; NA, not applicable

^a^ adjusted for age, sex, ethnicity, BMI, smoking, ischemic heart disease, hypertension, hyperlipidemia, and diabetes mellitus

The risk of RA was significantly elevated among patients with vitiligo as compared to controls (HR, 1.43; 95% CI, 1.03–1.98; *P* = 0.031). In sex- and age-stratified analyses, the risk of RA was prominently increased in males (HR, 1.90; 95% CI, 1.07–3.39; *P* = 0.029) and younger individuals (age < 32.4; HR, 2.25; 95% CI, 1.06–4.74; *P* = 0.034). The heightened risk of RA among patients with vitiligo retained its statistical significance after adjustment for demographics and comorbidities (fully-adjusted HR, 1.44; 95% CI, 1.02–2.02; *P* = 0.036; Table 2).

### The likelihood of vitiligo in patients with a preexisting diagnosis of RA

To investigate the likelihood of vitiligo subsequent to a history of RA, a case-control study design was conducted (Table 3). The development of subsequent vitiligo was significantly associated with a history of RA (OR, 1.66; 95% CI, 1.38–2.00; *P* < 0.001). In an age-stratified analysis, RA was found to predict vitiligo in all age categories as well as in females (OR, 1.76; 95% CI, 1.43–2.18; *P* < 0.001; Table 3).

**Table 3:** The odds of vitiligo in patients with a preexisting diagnosis of rheumatoid arthritis (case-control study design)

*N* (%) of preexisting rheumatoid arthritis in patients with vitiligo	148 (0.7%)
N (%) of preexisting rheumatoid arthritis in controls	440 (0.4%)
Unadjusted OR (95%CI) [p-value]	**1.66 (1.38-2.00) [< 0.001]**
*Sex- and age-stratified unadjusted analysis*	
Male-specific OR (95%CI) [p-value]	1.37 (0.93–2.03) [0.115]
Female-specific OR (95%CI) [p-value]	**1.76 (1.43–2.18) [< 0.001]**
≥ 32.4-year-old-specific OR (95%CI) [p-value]	**1.62 (1.33–1.98) [< 0.001]**
< 32.4-year-old-specific OR (95%CI) [p-value]	**2.05 (1.12–3.75) [0.017]**
*Multivariate adjusted analysis*	
Age- and sex-adjusted OR (95%CI) [p-value]	**1.66 (1.38–2.01) [< 0.001]**
Fully adjusted OR (95%CI) [p-value] ^a^	**1.67 (1.38–2.03) [< 0.001]**

**Bold**: significant value

*Abbreviations* N, number; OR, odds ratio; n, Number; CI, confidence interval; NA, not applicable

^a^ adjusted for age, sex, ethnicity, BMI, smoking, ischemic heart disease, hypertension, hyperlipidemia, and diabetes mellitus

In a multivariate analysis adjusting for putative confounders, a history of RA independently predicted a subsequent diagnosis of vitiligo (fully-adjusted OR, 1.67; 95% CI, 1.38–2.03; *P* < 0.001).

### Clinical characteristics of patients with vitiligo and comorbid RA

Table 4 describes the clinical features of patients with vitiligo and comorbid RA (*n* = 195) as compared to patients with vitiligo without RA (*n* = 20,656). The former group was typified by older mean (SD) age (56.4 [18.9] vs. 34.5 [22.3] years), female (OR, 3.01; 95% CI, 2.17–4.17, *P* < 0.001), and Jewish ethnicity (OR, 1.45; 95% CI, 1.02–2.07, *P* = 0.037) preponderance.

**Table 4:** Determinants of rheumatoid arthritis among patients with vitiligo

	Vitiligo with rheumatoid arthritis (*n* = 195)	Vitiligo without rheumatoid arthritis (*n* = 20,656)	OR (95% CI)	*p*-value
Age at the onset of vitiligo, years; mean (SD)^a^	56.4 (18.9)	34.5 (22.3)	**1.57 (1.46–1.68)** ^**a**^	**< 0.001**
Female sex, n (%)	147 (75.4%)	10,423 (50.5%)	**3.01 (2.17–4.17)**	**< 0.001**
Jewish ethnicity, n (%)	156 (80.0%)	15,155 (73.4%)	**1.45 (1.02–2.07)**	**0.037**
Obesity, n (%)	65 (33.3%)	4,090 (19.8%)	**2.03 (1.50–2.73)**	**< 0.001**
Smoking, n (%)	65 (33.3%)	5,142 (24.9%)	**1.51 (1.12–2.04)**	**0.007**
Ischemic heart disease, n (%)	32 (16.4%)	1,166 (5.6%)	**3.28 (2.24–4.82)**	**< 0.001**
Hyperlipidemia, n (%)	162 (64.6%)	6,162 (29.8%)	**4.30 (3.20–5.77)**	**< 0.001**
Hypertension	90 (64.2%)	3,268 (15.8%)	**4.56 (3.43–6.06)**	**< 0.001**
Diabetes mellitus, n (%)	59 (30.3%)	2,272 (11.0%)	**3.51 (2.58–4.78)**	**< 0.001**

**Bold**: significant values

*Abbreviations* n, number; SD, standard deviation

^a^ OR per 10-year increase in age

Patients with vitiligo and comorbid RA had higher frequency of comorbidities; smoking (OR, 1.51; 95% CI, 1.12–2.04; p-value 0.007), obesity (OR, 2.03; 95% CI, 1.50–2.73; *P* < 0.001), ischemic heart disease (OR, 3.28; 95% CI, 2.24–4.82; *P* < 0.001), hyperlipidemia (OR, 4.30; 95% CI, 3.20–5.77; *P* < 0.001), hypertension (OR, 4.56; 95% CI, 3.43–6.06, *P* < 0.001), and diabetes mellitus (OR, 3.51; 95% CI, 2.58–4.78, *P* < 0.001).

**Fig. 1:**
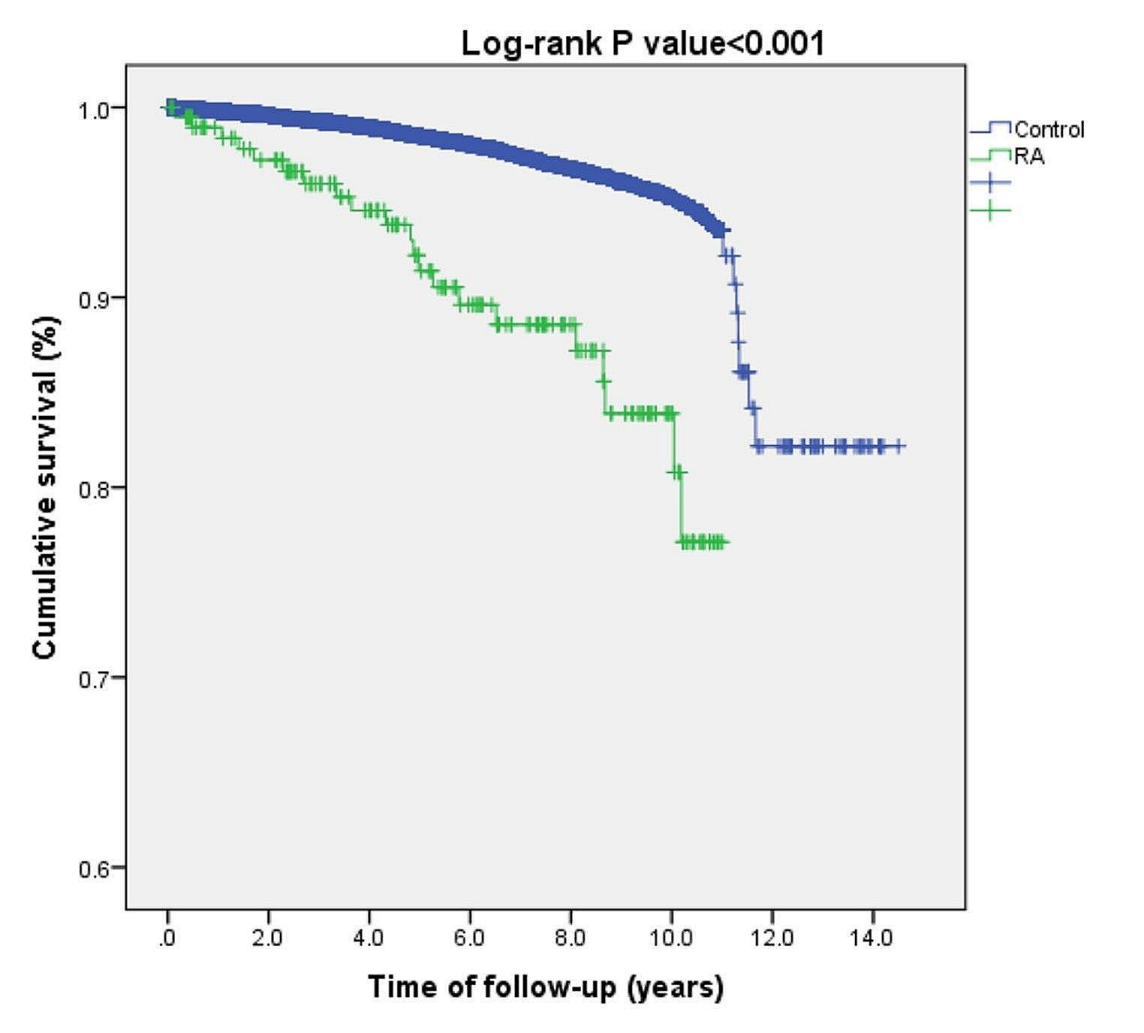
Kaplan-Meier curves demonstrating all-cause mortality of vitiligo patients with rheumatoid arthritis as compared to vitiligo patients without rheumatoid arthritis

We then carried out a survival analysis to evaluate the risk of all-cause mortality of vitiligo patients with comorbid RA relative to the remaining patients with vitiligo. Comorbid RA was associated with increased all-cause mortality in univariate (HR, 4.48; 95%CI, 2.89–6.94; *P* < 0.001; Fig. 1) and multivariate (fully-adjusted HR, 1.61; 95% CI, 1.03–2.51; *P* = 0.037) analyses.

## Discussion

The current population-based study sheds light on the existence of a bidirectional association between vitiligo and RA. Patients with vitiligo are at a 1.4-fold elevated risk of developing RA, and a preexisting diagnosis of RA predisposes individuals to have vitiligo by 1.7-fold. Patients with vitiligo and comorbid RA are characterized by a high burden of comorbidities and increased all-cause mortality.

Several epidemiological studies explored the relationship between vitiligo and RA (Supplementary Table 1). In their retrospective chart review following 2,441 patients with vitiligo, Sheth et al. found that 2.9% of vitiligo patients had RA, mostly in the African American/Black population [4]. Zhang et al. reported that RA was detected in 2.2% of their 5,601 patients with vitiligo, thus representing a 6.5-times increased frequency relative to the general population [13]. A 10-year retrospective cross-sectional study in the United States found that patients with vitiligo had a statistically significant increased prevalence of RA, affecting 1.6% of the study population [14]. While these studies threw an important spotlight on the association of vitiligo with RA, they were hampered by their cross-sectional design which interferes with the ability to draw conclusions regarding temporality and causality. Furthermore, most of these studies were characterized by small study populations. The coexistence of vitiligo and RA was additionally reported in individual patients by scattered case reports throughout the years [15–18].

The mechanism underlying the co-existence of vitiligo and RA has not been definitively established. However, vitiligo and RA share inflammatory pathways. Lee et al. [19] revealed an association between TNF-α polymorphisms and susceptibility to both RA and vitiligo. Specifically, their findings indicated that TNF-α polymorphism significantly elevates the risk of vitiligo among Middle Eastern populations.

JAK signaling pathway further supports the association between vitiligo and RA. Inflammatory cytokines play a crucial role in autoimmune responses, operating through the JAK-STAT pathway. The demonstrated effectiveness of JAK inhibitor therapy in treating RA implies their potential for addressing a broader range of immune-mediated conditions [20]. Vitiligo’s pathogenesis is characterized by IFN-γ, a key component of the immune response that leads to the destruction of melanocytes. JAK inhibitors can effectively target IFN-γ. Evidence from case series and studies suggests that JAK inhibitors offer promising vitiligo treatment [21]. Recently the FDA has approved Opzelura (ruxolitinib) a topical JAK inhibitor for treatment of nonsegmental vitiligo, and ongoing clinical trials further continuing [22].

Recent advancements in RA therapy include the use of TNF inhibitors, JAK inhibitors, and cytotoxic T-lymphocyte-associated antigen 4 (CTLA4) inhibitors. Recently, JAK inhibitors have emerged as a treatment for vitiligo. Utilizing these new drugs, originally developed for one disease, for the other disease, may simplify drug development and expand the therapeutic options for both diseases.

The current study provides valuable insights into the association between vitiligo and RA, utilizing a population-based study. Using the population-based CHS database, we ensure the accuracy of our estimates and minimize the risk of selection bias. The large CHS dataset used in our study includes 19,985 patients with vitiligo, which is one of the largest cohorts of vitiligo patients reported so far. This research bridges an existing gap in the field, as the absence of an extensive cohort study designed to practically investigate the association of vitiligo and rheumatoid arthritis. Our matching algorithm between case and control patients enables identical characteristics, further decreasing the risk of bias. The limitations of our study include the clinical, rather than histological, diagnosis of vitiligo. In some cases, the presentation of vitiligo may be atypical or less apparent, leading to potential inaccuracies in vitiligo diagnosis.

In conclusion, the current study confirms a significant bidirectional association between vitiligo and RA. Patients with vitiligo and comorbid RA experience a high burden of cardiometabolic comorbidities and are at a significantly elevated hazard of death. Our findings might raise awareness about the shared immunological mechanisms and potential therapeutic avenues involving JAK inhibitors.

## Electronic supplementary material

Below is the link to the electronic supplementary material.

Supplementary Material 1

## Data Availability

No datasets were generated or analysed during the current study.
