# Supplementary material for: A bidirectional autoimmune cluster between vitiligo and rheumatoid arthritis: a large-scale population-based study
Source: Arch Dermatol Res. 2024 Jun 8;316(7):366. doi: 10.1007/s00403-024-02965-7 (PMC11162386; doi:10.1007/s00403-024-02965-7)
Supplement: Supplementary file 1 — Supplementary Material 1 [file 403_2024_2965_MOESM1_ESM.docx]

| Comments | OR  (95%CI) | % of RA in the control group | % of RA in patients with vitiligo | Number of patients with vitiligo | Publication type | Title | Publication year | Author |
| --- | --- | --- | --- | --- | --- | --- | --- | --- |
| Vitiligo patients had a 6.5-fold increased prevalence of rheumatoid arthritis, compared to the general population (p- value < 0.01) | NA | 0.32–0.34%^a^ | 2.20% | 5,601 | Cross-sectional retrospective study | The analysis of genetics and associated autoimmune diseases  in Chinese vitiligo patients | 2009 | Zheng Zhang |
|  | NA | NA | 2.9% | 2,441 | Cross sectional retrospective study | Comorbidities Associated with Vitiligo:  A Ten-Year Retrospective Study | 2014 | Vaneeta M. Sheth |
| OR is only significantly associated with vitiligo in females aged between 60–79 years of age. | 1.97 (1.22–3.16) | NA | 0.40% | 14, 883 | Cross-sectional retrospective study | Comorbidity profiles in association with vitiligo: a nationwide population-based study in Taiwan | 2014 | Y.T. Chen |
| The study population identified during 2009–2013. | 1.342 (1.054–1.710) | 0.10% | 0.13% | 86,210 | Cross-sectional study | Increased risk of comorbid rheumatic disorders in vitiligo patients: A nationwide population-based study | 2017 | Chong Won Choi |
| Vitiligo patients had a 2.14-fold increased prevalence of rheumatoid arthritis, compared to the general population (p- value < 0.01) | NA | National prevalence  per 100,000 population- 753 | 1.6% ^b^ | 1,487 | Cross-sectional retrospective study | Comorbid diseases of vitiligo: A 10-year  cross-sectional retrospective study of an urban US population | 2020 | Ali Hadi |

**Supplementary Table 1**: Literature review of the association between vitiligo and rheumatoid arthritis

^a^ population prevalence data utilized from another study.

^b^ disease prevalence was extrapolated to prevalence in 100,000 patients- 1614
